# Does Prop-2-ynylideneamine, HC≡CCH=NH, Exist in Space? A Theoretical and Computational Investigation

**DOI:** 10.3390/ijms150611064

**Published:** 2014-06-19

**Authors:** Osman I. Osman, Shaaban A. Elroby, Saadullah G. Aziz, Rifaat H. Hilal

**Affiliations:** 1 Chemistry Department, Faculty of Science, King Abdulaziz University, P.O. Box 80203, Jeddah 21589, Saudi Arabia; E-Mails: skamel@kau.edu.sa (S.A.E.); saziz@kau.edu.sa (S.G.A.); rhilal@kau.edu.sa (R.H.H.); 2 Chemistry Department, Faculty of Science, University of Khartoum, P.O. Box 321, Khartoum 11111, Sudan; 3 Chemistry Department, Faculty of Science, Benisuief University, Benisuief 6251, Egypt; 4 Chemistry Department, Faculty of Science, Cairo University, Cairo 12613, Egypt

**Keywords:** Z-prop-2-ynylideneamine, tautomerization, thermodynamic, Interstellar Medium, DFT, MP2, CCSD, NBO

## Abstract

MP2, DFT and CCSD methods with 6-311++G** and aug-cc-pvdz basis sets have been used to probe the structural changes and relative energies of E-prop-2-ynylideneamine (I), Z-prop-2-ynylideneamine (II), prop-1,2-diene-1-imine (III) and vinyl cyanide (IV). The energy near-equivalence and provenance of preference of isomers and tautomers were investigated by NBO calculations using HF and B3LYP methods with 6-311++G** and aug-cc-pvdz basis sets. All substrates have Cs symmetry. The optimized geometries were found to be mainly theoretical method dependent. All elected levels of theory have computed I/II total energy of isomerization (Δ*E*) of 1.707 to 3.707 kJ/mol in favour of II at 298.15 K. MP2 and CCSD methods have indicated clearly the preference of II over III; while the B3LYP functional predicted nearly similar total energies. All tested levels of theory yielded a global II/IV tautomerization total energy (Δ*E*) of 137.3–148.4 kJ/mol in support of IV at 298.15 K. The negative values of Δ*S* indicated that IV is favoured at low temperature. At high temperature, a reverse tautomerization becomes spontaneous and II is preferred. The existence of II in space was debated through the interpretation and analysis of the thermodynamic and kinetic studies of this tautomerization reaction and the presence of similar compounds in the Interstellar Medium (ISM).

## 1. Introduction

The chemistry of transient compounds that contain carbon-nitrogen double bonds, that is, imines [1], has recently caught the attention of many investigators [2,3,4]. This is mainly due to their instability in normal conditions, and hence act as important intermediates in many organic reactions [5,6]. As a result they are thought to play remarkable roles in the chemistry of interstellar compounds [7]. 

The first member of the imine series, methyleneimine, CH_2_=NH, was first discovered by low temperature matrix IR spectroscopy [8]. A later detection by microwave spectroscopy [9] has facilitated its revelation in Sgr B2 as an ample interstellar compound [10]. Of particular interest to our study is the highly transient ketenimine, CH_2_=C=NH, that has been characterized by IR [11] and microwave spectroscopy [12] and then very recently detected also in Sagittarius B2(N) [Sgr B2(N)] Hot Cores [13]. It is noteworthy that ketenimine is a tautomer of methyl cyanide, CH_3_C≡N. The latter molecule is extremely stable compared to the former (by *ca.* 32 kcal/mol [14]). As a result methyl cyanide was characterized by microwave spectroscopy [15] as early as 1950. Its early detection in space [16,17] was recently confirmed by its strong presence toward the Hot Core Regions W51e1/e2 [18]. Methyl isocyanide, CH_3_N≡C, their third isomer, has also been detected in space [19] with an abundance ratio CH_3_N≡C/CH_3_C≡N of *ca.* 0.05. It is striking that the observation of isomers in space has become more common, especially with large molecules. With the exception of diatomic molecules, of the 135 molecules detected in space till 2005, *ca.* 30% of them have their isomeric rivals characterized as interstellar compounds [20]. 

Prop-2-ynylideneamine, HC≡CCH=NH, is a transient tautomer of vinyl cyanide, CH_2_=CHC≡N. The latter molecule is very well characterized in the laboratory [21,22,23,24,25] and space [26,27]. The two isomers, Z-prop-2-ynylideneamine and E-prop-2-ynylideneamine, were detected by microwave [28,29,30] and infrared [31,32] techniques. Z-prop-2-ynylideneamine was shown experimentally to be more stable than its E-isomer by 0.78 ± 0.2 kcal/mol [29]. Our previous theoretical study on these two imine isomeric forms [33] confirmed this estimation and prompted the present contribution as a response to a suggestion of one of the reviewers. His/her suggestion originated from the search for Z-prop-2-ynylideneamine in Taurus Molecular Cloud 1 (TMC1) that provided an upper limit for its abundance [34], as compared to its isomeric form, vinyl cyanide, which proved to be an abundant interstellar species in Sagittarius B2(N) Hot Cores (Sgr B2(N)) [26,27]. 

In this contribution we endeavour to investigate theoretically and computationally the aspects of the tautomerization reaction of prop-2-ynylideneamine and vinyl cyanide through a 1,3-proton migration [35]. In particular, we aim to learn the molecular structures and energetics of the reactants, Transition States (TS), intermediate and products involved in these isomerization and tautomerization processes. We intend also to understand these molecular properties by Natural Bond Orbital (NBO) investigations. We have done so by calculating: (1) the geometries of these isomers and tautomers; (2) the free energy changes or the equilibrium constants of the isomerization and the tautomerization reactions; and (3) the delocalization energies of the isomers and tautomers. These calculations have two functions. First, they enable the comparison of the results obtained from the different theoretical methods. Second, they predict the stabilities and reaction pathways for these isomers and tautomers and hence shed light on their possible existence in ISM. 

## 2. Results and Discussion

### 2.1. Molecular Structure

**Figure 1:**
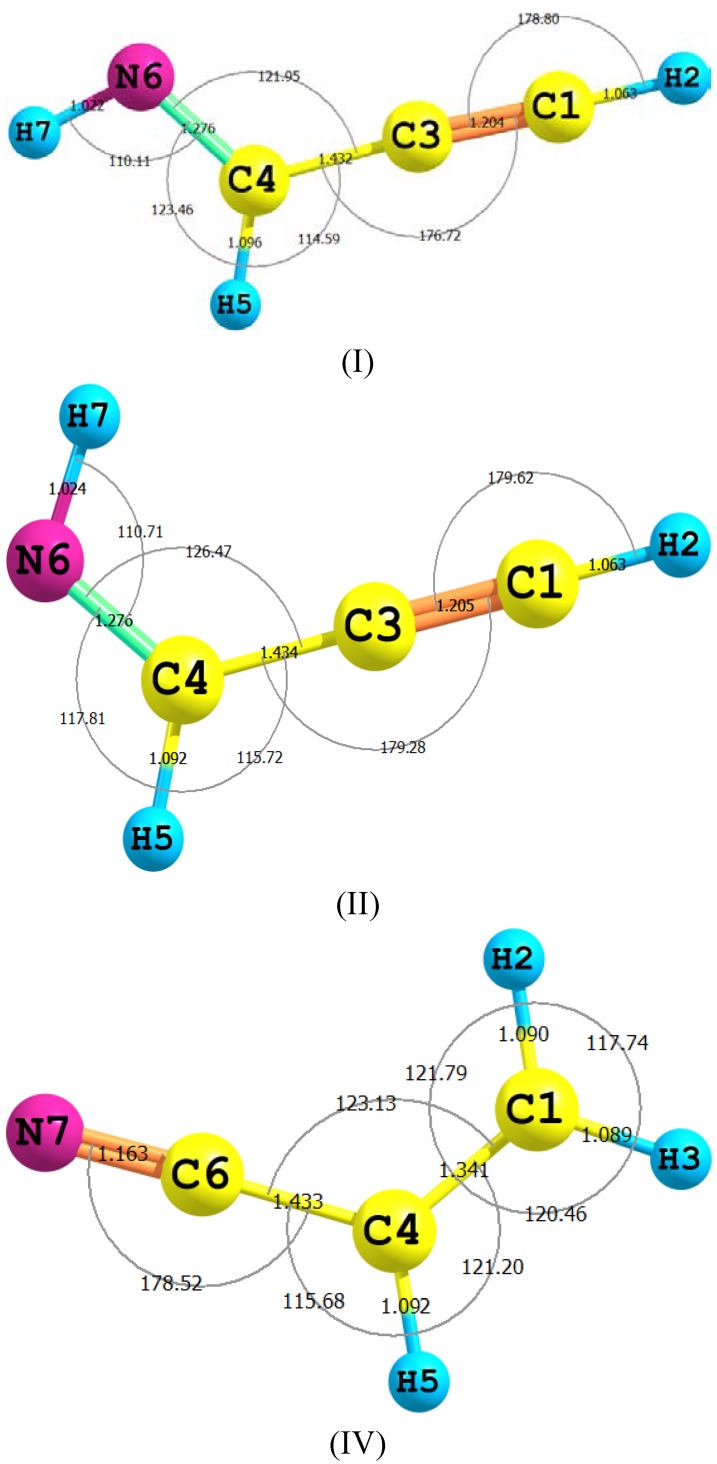
The numbering of atoms for E-prop-2-ynylideneamine (I), Z-prop-2-ynylideneamine (II) and vinyl cyanide (IV), together with their optimized bond lengths (Å) and angles (degrees) using B3LYP/aug-cc-pvdz level of theory.

Figure 1 depicts the optimized geometries of E-prop-2-ynyideneamine (I), Z-prop-2-ynylideneamine (II), and vinyl cyanide (IV) computed by using B3LYP/aug-cc-pvdz level of theory. In Table 1 and Table 2 are listed the optimized geometrical parameters and dipole moments of I, II and IV which were estimated by using MP2, B3LYP and CCSD methods with 6-311++G** and aug-cc-pvdz basis sets. The experimental microwave data and dipole moments for II [29,30] and IV [21,24] are included for comparison purposes. The CCSD results should be excellent estimates for these substrates and therefore be extremely valuable in the analysis of their experimental data therein. In addition, the MP2 and B3LYP data can be compared with that from CCSD and hence allows the assessment of using MP2 and B3LYP methods for studying larger imine compounds.

All the substrates studied possess Cs symmetry. Their optimized bond lengths and angles obtained by using all levels of theory were found to be consistent. Generally, irrespective of the basis set used, the B3LYP bond lengths are shorter and the bond angles are larger than those from CCSD. This is in complete agreement with the data obtained for the phosphalkenes [36]. The MP2 data are comparable with that from CCSD method. As for the basis sets, aug-cc-pvdz yielded longer bond lengths and smaller bond angles compared to the 6-311++G** values; regardless of the applied theoretical method. The largest effects for bond lengths and angles are obtained by using MP2 method (C≡C bond length varies by *ca.* 0.015 Å while the CNH angle by *ca.* 0.32°). This indicates that the MP2 values are relatively scattered compared to those of the B3LYP and CCSD methods. 

All our elected levels of theory have consistently reproduced more or less the experimental bond lengths and angles of II [30] and IV [24]. We can thus safely conclude from Table 1 and Table 2 that their accuracies are comparable. As for the calculated dipole moments, the CCSD method has yielded the relatively most accurate values compared to those of the B3LYP and MP2 methods.

**Figure 2:**
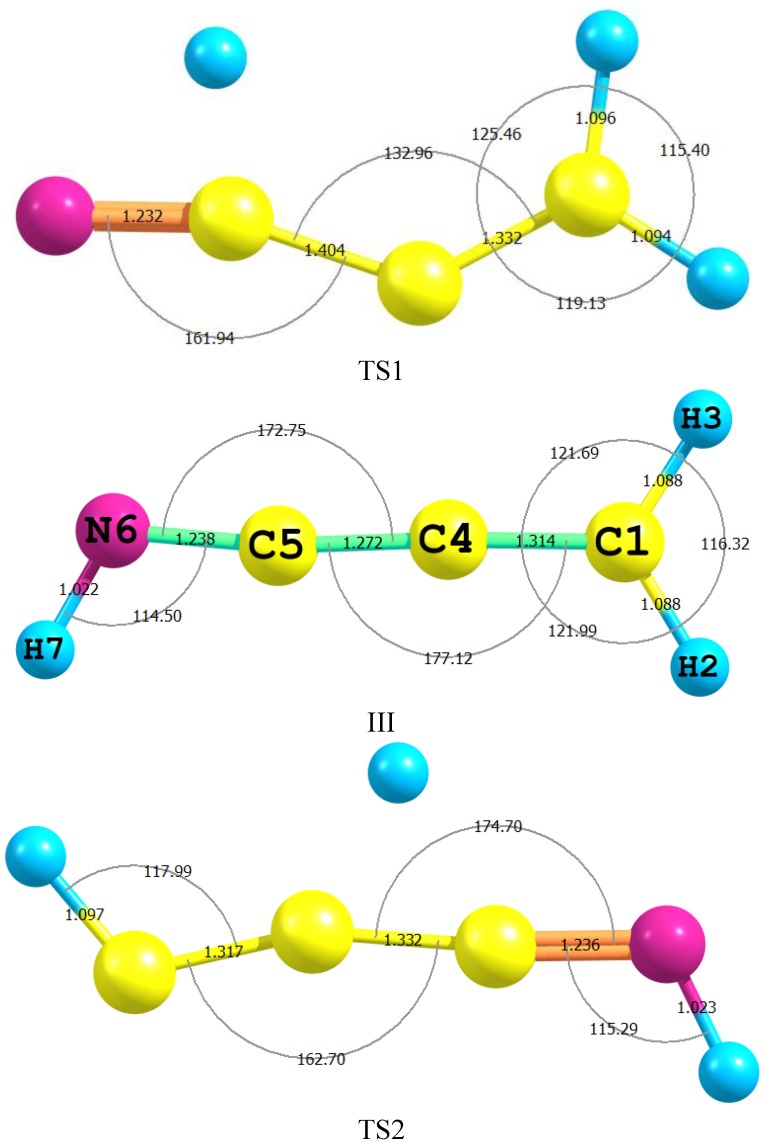
The numbering of atoms for the Intermediate (III) and Transition States (TS1 and TS2) together with their optimized bond lengths (Å) and angles (degrees) using B3LYP /aug-cc-pvdz level of theory.

Table 3 lists some selected geometrical parameters of II, TS1, III, TS2 and IV, which were calculated by applying B3LYP/aug-cc-pvdz level of theory. The experimental geometrical parameters for II [30] and IV [22] are also included for comparison purposes. The optimized geometries of TS1, III and TS2 are shown in Figure 2. In the route for the interconversion between II and IV; the intermediate (III) is connected to II through TS2 and to IV via TS1. 

As shown in Figure 1 and Table 3, the C4–H5 bond in II of 1.092 Å has elongated in TS2 to become 1.433 Å; as a step for a 1,3-proton migration to form C1–H3 of III (1.088 Å). This process is accompanied by the shortening of the C3–C4 bond of 1.434 Å in II to become 1.332 Å in TS1 and to settle at 1.272 Å in III. This trend reflects the tendency for a conversion from a single to a double bond character. These changes can also be visualized in conjunction with two bond angle changes: first, the opening up of the CCN of 126.5° in II to turn into 174.7° in TS2 and finally to stabilize at 172.75° in III; and second, the closing up of the C≡CH bond of II by *ca.* 62° to form TS2 and to open up again by 3.7° to brew III. These proclivities are in excellent agreement with similar theoretical studies [33].

**Table 1:** The optimized parameters (bond lengths/Å and bond angles/degrees) and dipole moments (μ/Debye) of E-HC≡CCH=NH (I) and Z-HC≡CCH=NH (II) using MP2, B3LYP and CCSD methods with 6-311++G** and aug-cc-pvdz basis sets.

Method	Basis Set	H-C	C≡C	C-C	C=N	NH	CH	CCN	CNH	µ/Debye
MP2	6-311++G**	1.065	1.221	1.438	1.288	1.024	1.095	120.95	108.61	2.27
	aug-cc-pvdz	1.075	1.236	1.445	1.297	1.029	1.102	120.79	108.58	2.17
B3LYP	6-311++G**	1.063	1.204	1.432	1.276	1.022	1.096	121.95	110.11	2.07
	aug-cc-pvdz	1.070	1.214	1.437	1.281	1.024	1.102	121.67	109.90	1.97
CCSD	6-311++G**	1.066	1.212	1.448	1.281	1.023	1.095	121.13	109.11	2.05
	aug-cc-pvdz	1.076	1.225	1.457	1.289	1.028	1.102	121.00	109.06	1.97
Experimental ^a^		-	-	-	-	-	-	-	-	1.90
MP2	6-311++G**	1.066	1.222	1.440	1.289	1.025	1.091	125.79	108.82	2.58
	aug-cc-pvdz	1.075	1.237	1.448	1.297	1.031	1.096	125.76	109.14	2.44
B3LYP	6-311++G**	1.063	1.205	1.434	1.276	1.023	1.091	126.45	110.71	2.41
	aug-cc-pvdz	1.070	1.215	1.439	1.281	1.026	1.097	126.37	110.51	2.31
CCSD	6-311++G**	1.067	1.212	1.452	1.281	1.024	1.091	125.68	109.52	2.32
	aug-cc-pvdz	1.076	1.226	1.460	1.288	1.029	1.100	125.68	109.75	2.22
Experimental ^b^		1.057	1.207	1.431	1.286	1.039	1.101	125.38	108.89	2.15

^a^ Experimental dipole moment of I taken from [29]; ^b^ microwave substitution structure of II taken from [30] and experimental dipole moment of II taken from [29]. The first seven lines of data are for I; the second seven lines of data are for II.

**Table 2:** The optimized bond lengths (Å) and bond angles (deg.) of CH_2_=CHC≡N (IV) using MP2, B3LYP and CCSD methods with 6-311++G** and aug-cc-pvdz basis sets.

Method Basis Set	MP2		B3LYP		CCSD		Expt. ^a,b^
	6-311++G**	aug-cc-pvdz	6-311++G**	aug-cc-pvdz	6-311++G**	aug-cc-pvdz	
C-H^t^	1.085	1.092	1.083	1.089	1.086	1.093	1.097
C-H^c^	1.084	1.092	1.083	1.090	1.086	1.094	1.093
C=C	1.344	1.353	1.335	1.341	1.342	1.351	1.343
C-H^u^	1.086	1.093	1.085	1.092	1.086	1.094	1.085
C-C	1.435	1.443	1.428	1.433	1.445	1.454	1.429
C≡N	1.177	1.189	1.156	1.163	1.162	1.172	1.160
CCH^t^	120.28	120.17	120.56	120.46	120.4	120.4	118.5
CCH^c^	121.44	121.32	121.79	121.78	121.7	121.6	120.3
C=CH^u^	121.39	121.31	121.24	121.21	121.9	121.8	121.6
CCC	122.11	122.20	123.11	123.11	122.1	122.1	122.2
CCN	179.05	179.10	178.72	178.46	179.0	179.0	178.4
µ/Debye	4.47	4.51	4.05	4.04	3.90	3.95	3.92

^a^ Microwave substitution structure taken from [24]; ^b^ Experimental dipole moment taken from [25].

The most remarkable changes that link III to IV through TS1 include: first, the shortening of the C1–C3 bond, in IV, and then of 1.431 Å to become 1.332 and 1.314 Å in TS1 and III respectively. That is, it was transferred from a single to a double bond; Second, the CN triple bond in IV of 1.163 Å was elongated to form a typical double bond (1.238 Å) through that of TS1 of 1.232 Å; Third, the roughly linear CCN bond of IV was bent by *ca.* 18.1° and 7.2° in TS1 and III, respectively. This theoretical perception of the mechanism of the interconversion between II and IV will also be addressed adequately by Natural Bond Orbital (NBO) approach in a later section. 

**Table 3:** Optimized parameters (bond lengths in Å and bond angles and dihedral angles in degrees of Z-HC≡CCH=NH(II) ^a^, Transition States (TS1 and TS2), the Intermediate, CH_2_=C=C=NH(III) and CH_2_=CHC≡N(IV) ^b^ which have been calculated using B3LYP/aug-cc-pvdz level of theory.

Parameter	II	TS2	III	TS1	IV
H2–C1	1.063 (1.057)	1.097	1.088	1.095	1.090 (1.089)
C1–C3	1.205 (1.207)	1.317	1.314	1.332	1.341 (1.343)
C3–C4	1.434 (1.431)	1.332	1.314	1.332	1.433 (1.429)
C4–N6	1.276 (1.286)	1.236	1.238	1.232	1.163 (1.163)
N6–H7	1.024 (1.039)	1.023	1.022	1.585	-
C4–H5	1.092 (1.101)	1.433	-	1.096	1.092(1.086)
C4C5N6	126.5 (125.3)	174.7	172.75	161.9	178.4(178.4)
C5N6H7	110.7 (108.9)	45.8	114.2	45.8	-
H3C1N7H9	180.00	−179.9	0.00	−179.9	-
C4C1N7H9	−0.004	−179.9	180.0	−179.9	-

Values between parentheses are: ^a^ taken from [30]; ^b^ taken from [22].

### 2.2. Isomerism and Tautomerism

In Table 4 are shown the B3LYP, MP2 and CCSD methods with 6-311++G** and aug-cc-pvdz basis sets values of Δ*E*, Δ*H*, Δ*S*, Δ*G* and *K* for the isomerization reaction of I and II. All levels of theory have indicated clearly that II is favoured by total energy differences (Δ*E*) of 1.707 to 3.707 kJ/mol, in good agreement with the experimental value of 3.264 ± 0.837 kJ/mol [29]. Comparing the results from the six levels of theory, one can assess the effects of the methods and the basis sets. A global look at the results in Table 4 shows clearly that Δ*E*, Δ*H*, Δ*S* and Δ*G* are both basis set and theoretical method dependent. For the aug-cc-pvdz basis set, the effect of the theoretical method is minimal. This can be visualized by the closeness of values. It can be seen clearly that the B3LYP functional gave comparable values, followed by the CCSD method; while MP2 method yielded relatively far apart ones.

**Table 4:** MP2, B3LYP and CCSD methods with 6-311++G** and aug-cc-pvdz basis sets zero-point Reaction Energies, Enthalpies, Entropies, Free Energies Changes and Equilibrium Constants at 298.15 K for the equilibrium E-HCCCH=NH ↔ Z-HCCCH=NH.

Method	Basis Set	Δ*E* kJ/mol	Δ*H* kJ/mol	Δ*S* J/mol.K	Δ*G* kJ/mol	*K*
MP2	6-311++G**	−2.046	−2.138	−0.282	−2.054	2.291
	aug-cc-pvdz	−3.707	−3.452	+2.484	−4.192	5.425
B3LYP	6-311++G**	−2.920	−2.958	+0.364	−3.067	3.445
	aug-cc-pvdz	−3.063	−2.954	+1.375	−3.364	3.885
CCSD	6-311++G**	−1.707	−1.757	+0.126	−1.795	2.063
	aug-cc-pvdz	−3.084	−2.887	+2.006	−3.485	4.080

It is worth mentioning that 6-311++G** basis set gave smaller estimates of ∆*E*, ∆*H*, ∆*S*, ∆*G* and *K* values compared to those from aug-cc-pvdz using all three methods. The former basis set gave a reaction energy change (∆*E*) with errors of 37% (MP2), 10% (B3LYP) and 47% (CCSD) compared to the experimental value of 3.264 ± 0.837 kJ/mol [29]. The latter basis set yielded relatively small errors: 13% (MP2), 6% (B3LYP) and 5% (CCSD). These large errors, can be explained in terms of the inaccuracy (with an error of the order of 25.5%) of their experimental energy difference of 3.264 ± 0.837 kJ/mol [29]. 

Apart from MP2/6-311++G**; all levels of theory yielded positive Δ*S* values, which indicate that II is favoured at all temperatures. A negative Δ*S* value shown by MP2/6-311++G** signalizes II preference at low temperature, but favours I at high temperature. At 298.15 K, ∆*G* is highly overpowered by ∆*H*, with little contribution (in most cases less that 10%) from *T*∆*S*. The *K* values indicate that the equilibrium concentration of II ranges from double to five times that of I. It is interesting to note that *K* is sensitive to ∆*G*; a change of 1 kJ/mol at 298.15 K has affected *K* by a factor of more than one. 

Table 5 lists ∆*E*, ∆*H*, ∆*S*, ∆*G* and *K* values for the equilibrium tautomerization reaction between Z-prop-2-ynylidemenamine (II) and vinyl cyanide (IV) using MP2, B3LYP and CCSD methods with 6-311++G** and aug-cc-pvdz basis sets. The negative values of Δ*S* denote that IV is favoured at low temperature by a total energy difference (Δ*E*) of 137.3 to 148.4 kJ/mol. Thus at high temperature, a reverse reaction becomes spontaneous and hence II is preferred. This might indicate that II could be present in hot core forming stars. The values of *K* are indicative of the huge equilibrium concentration of IV compared to that of II. These results confirmed the high ISM abundance of IV [26,27] and the equivocal existence of II [34] in space. A striking remark is that all levels of theory gave consistent and comparable values. The MP2 and CCSD methods at both basis sets computed comparable ∆*E*, ∆H and ∆*G* values which deviate from the B3LYP method values by *ca.* 10 kJ/mol. The aug-cc-pvdz values of ∆*S* are approximately twice those of 6-311++G** basis set.

**Table 5:** MP2, B3LYP and CCSD methods with 6-311++G** and aug-cc-pvdz basis sets zero-point Reaction Energies, Enthalpies, Entropies, Free Energies Changes and Equilibrium Constants at 298.15 K for the equilibrium Z-HCCCH=NH ↔ CH_2_=CHC≡N.

Method	Basis Set	Δ*E* kJ/mol	Δ*H* kJ/mol	Δ*S* J/mol.K	Δ*G* kJ/mol	*K*
MP2	6-311++G**	−147.507	−148.189	−4.042	−146.984	5.648 × 10^25^
	aug-cc-pvdz	−147.436	−148.499	−6.571	−146.540	4.723 × 10^25^
B3LYP	6-311++G**	−138.449	−138.674	−2.130	−138.039	1.530 × 10^24^
	aug-cc-pvdz	−137.336	−138.076	−4.236	−136.813	9.333 × 10^23^
CCSD	6-311++G**	−147.535	−148.073	−3.029	−147.170	6.088 × 10^25^
	aug-cc-pvdz	−148.375	−149.304	−5.618	−147.629	7.326 × 10^25^

### 2.3. Activation Energies

**Figure 3:**
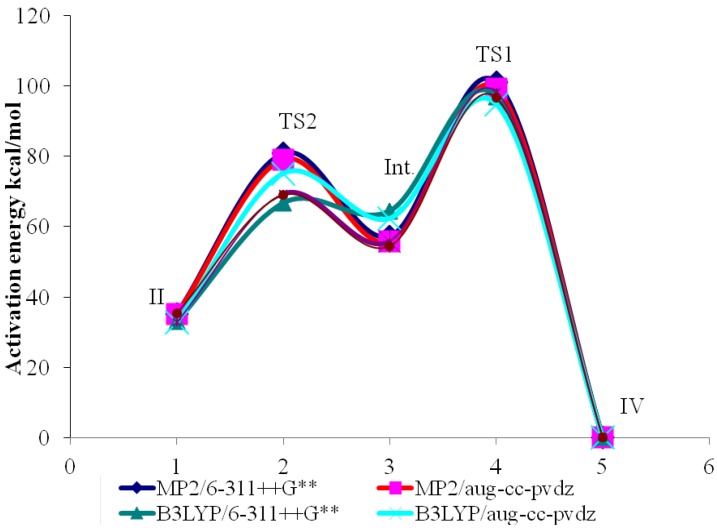
Schematic potential energy profile showing the activation energies (kcal/mol) for the tautomerization of II and IV, which were calculated by using the elected levels of theory.

The zero-point electronic and activation energies of the tautomerization reaction of Z-prop-2-ynylidineamine (II) and vinyl cyanide (IV) through two transitions states (TS1 and TS2) and an intermediate (prop-1,2-diene-1-imine, CH_2_=C=C=NH) (III) using MP2, B3LYP and CCSD methods with 6-311++G** and aug-cc-pvdz basis sets are listed in Table 6 and shown graphically in Figure 3. The elected levels of theory have estimated relative total energy differences (Δ*E*) of 33.3–35.5 kcal/mol in preference of vinyl cyanide (IV) over Z-prop-2-ynylideneamine (II). This result is in excellent agreement with a recent theoretical study [33]. 

On the one hand, III was predicted to be less stable than II by 8.53 kcal/mol (MP2/6-311++G**), 8.29 kcal/mol(MP2/aug-cc-pvdz), 7.17 kcal/mol (CCSD/6-311++G**) and 6.91 kcal/mol (CCSD/aug-cc-pvdz). Surprisingly, the B3LYP functional values show the competiveness of III by narrowing the gap up to 0.32 kcal/mol (6-311++G**) and 0.21 kcal/mol (aug-cc-pvdz) in preference of II. These findings indicate that the MP2 and CCSD methods gave comparable values which unequivocally favour II; whilst those of the B3LYP functional indicated that the two tautomers have nearly similar energies and hence casts some doubt about the predilection of II. On the other hand, all elected levels of theory computed IV to be favoured over II by not less than 33 kcal/mol and hence strongly assured the vantage of IV.

**Table 6:** Zero-point electronic energies (a.u.) and activation energies (kcal/mol) of the tautomerization of Z-prop-2-ynylidineamine (HC≡CCH=NH) (II) to form vinyl cyanide (CH_2_=CHC≡N) (IV) through the Transition State 1 (TS1), (prop-1,2-diene-1-imine, CH_2_=C=C=NH) (III) and Transition State 2 (TS2).

Method	Basis Set	II	TS1	III	TS2	IV
MP2	6-311++G**	−170.27730	−170.148485	−170.263702	−170.172449	−170.33348
	Activ. Energy	80.83	-	57.26	-	101.05
	aug-cc-pvdz	−170.24374	−170.117638	−170.230527	−170.141694	−170.29989
	Activ. Energy	79.13	-	55.74	-	99.27
B3LYP	6-311++G**	−170.82975	−170.723374	−170.829238	−170.728235	−170.88290
	Activ. Energy	66.75	-	63.38	-	97.05
	aug-cc-pvdz	−170.80018	−170.696274	−170.799846	−170.702204	−170.85343
	Activ. Energy	65.20	-	61.27	-	94.90
CCSD	6-311++G**	−170.29782	−170.18736	−170.286399	−170.197429	−170.35401
	Activ. Energy	69.31	-	55.83	-	98.26
	aug-cc-pvdz	−170.26630	−170.15642	−170.255619	−170.168856	−170.32282
	Activ. Energy	68.95	-	54.44	-	96.61

Figure 3 depicts the energy profiles plots that connect the reactant to product through first-order saddle points and a metastable intermediate using MP2, B3LYP and CCSD methods with 6-311++G** and aug-cc-pvdz basis set. A general trend is observed from all methods. However, the B3LYP functional gave lower energies for all substrates compared to those of MP2 and CCSD methods, and, in addition, computed the intermediate (III) to have a slightly higher total energy relative to II. The analysis of the normal mode of TS2 imaginary frequency (−1053 cm^−1^) revealed displacement of C1–H3 bond length of III, in 1,3-hydrogen shift [35,37], to produce II; while that of the normal mode of TS1 imaginary frequency (−1080 cm^−1^) showed the N6–H7 bond length displacement, through another 1,3-proton sift, to generate IV. These mechanistic routes confirmed correctly the linkage between the modeled reactant and product.

As is shown in Figure 3, all our elected levels of theory exhibited that the activation barriers for the tautomerization reaction between II and IV (*ca.* 65–81 kcal/mol) are lower than those of the reverse reaction (95–101 kcal/mol). This result is in excellent agreement with a similar theoretical study [33]. It means that IV is extremely favoured. Generally, the magnitudes of the activation energies are both theoretical method and basis set dependent; *i.e.*, they follow the order: MP2 > B3LYP > CCSD for the theoretical methods and 6-311++G** > aug-cc-pvdz for the basis sets [33,38]. 

To the best of our knowledge, no experimental or theoretical investigation have been devoted to elucidate the mechanistic route that led to the high abundance of IV [26] in Sgr B2(N) Hot Core and the dubitable existence of II [34] in TMC1 as a cold dark cloud. The former ridge has a temperature of 300 K and a low pressure in dense (3 × 10^3^ cm^−3^) star-forming regions [39], whilst Pratap *et al.* [40] have shown that the latter cloud is cold (*ca.* 10 K) with a relatively high density of 8 × 10^4^ cm^−3^. This means that II could show up in Sgr B2(N), as a relatively hot cloud, with detectable abundance. This is in excellent agreement with our results from all elected levels of theory, which predicted the preference of II over IV at high temperature. This conjecture could also be supported by the detection of ketenimine in Sgr B2(N) as a result of methyl cyanide tautomerization [13]. The recent revelation of E-cyanomethanimine (isoelectronic with II) in Sgr B2(N) by Zaleski *et al.* [41] consolidates further our earlier intuition; that II is highly likely to be existent in Sgr B2(N) despite its ambiguous presence in TMC1 [34]. 

Our preference of II over I to be present in Sgr B2(N) rests on two reasons. First, all our calculations have shown that the equilibrium concentration of II is twice to five times that of I. Second, Sugie *et al.* [29] have shown that the experimental total dipole moment of II is slightly greater than that of I by *ca.* 13%. However, this is not to exclude totally the possibility of observing I in Sgr B2(N) as both isomers are of comparable energies [29]. This anticipation is recently supported by the detection of the Z- and E-isomers of ethanimine in both the laboratory and Sgr B2(N) [42].

It is noteworthy that global potential energy minima have been reached for III using all elected levels of theory. Nevertheless, and to the best of our knowledge, prop-1,2-diene-1-imine (III) has never been isolated and detected by any spectroscopic method, nor been studied theoretically. Relying on these findings, we conjecture that this metastable species could be isolated and characterized in the laboratory as a prerequisite for its highly possible presence in space. A full investigation of the theoretical and computational characterization of III will be addressed in our upcoming contribution.

### 2.4. Natural Bond Orbital (NBO) Analyses

**Figure 4:**
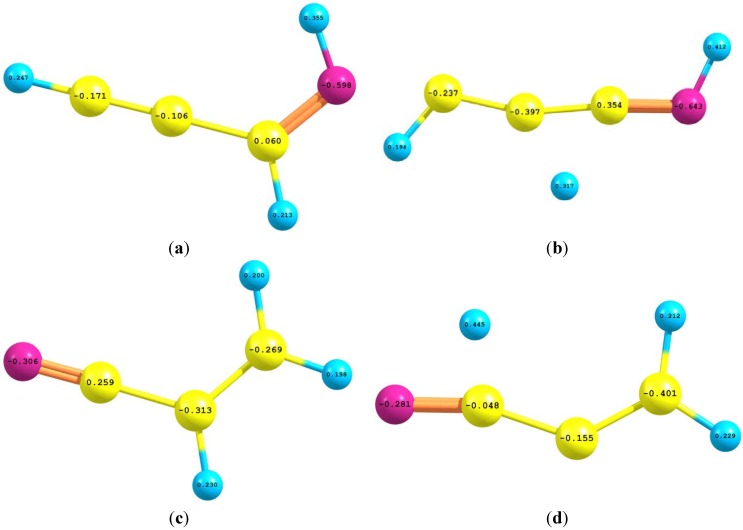
The NBO charge distribution of (**a**) Z-prop-2-ynylideneamine (II); (**b**) TS2; (**c**) vinyl cyanide (IV) and (**d**) TS1 which were calculated using B3LYP/aug-cc-pvdz level of theory. The migrating hydrogen atom acquired a positive charge (+0.213e) in II and an intensified charge (+0.317e) in TS2 while it acquired a positive charge (+0.230e) in IV that was condensed (+0.445e) in TS1.

Figure 4 depicts the natural atomic charges of II, TS2, IV and TS1 which were calculated by using B3LYP/aug-cc-pvdz level of theory. It is noticeable that the positive charge disperses amongst the hydrogen atoms of II and IV and does not localize on the future migrating H-atom that carried positive charges of +0.213e and +0.230e in II and IV respectively. By analogue, this leaving hydrogen atom had acquired a much more positive charge in TS1 and TS2 of +0.317e and +0.445e respectively.

In Table 7 are listed the second order perturbation (*E*_(2)_) computation of the hyperconjugative energies of II, III, IV, TS1 and TS2 which were estimated by using B3LYP/aug-cc-pvdz level of theory. Natural Bond Orbital (NBO) theory [43,44] has been widely accepted for analyzing hyperconjugative interactions [45] by using second order perturbation (*E*_(2)_) energies given by the relation:

*E*_(2)_ = Δ*E_ij_* = q*_i_* (F*_ij_*)^2^/Δε

where *q_i_* is the occupancy of the donor orbital, *F_ij_* is the off-diagonal elements of the NBO Kohn-Sham Matrix and Δε is the energy difference between a donor orbital (*i*) and an acceptor orbital (*j*). Referring back to Table 7, we can make the following observations: (1) The most influential hyperconjugative interaction for II, III and IV is the π_C1C3_ → π*_C4N6_ that stabilized each one of them by 16.02, 33.45 and 18.11 kcal/mol, respectively. This comparatively high delocalization energy for III, almost double those for II and IV, is indicative of its CCCN moiety nearly linear nature, which facilitates a stronger overlapping. Hence, this extremely favourable vicinal π → π* overlap has shortened the C3=C4 bond length of III by 0.035 Å, compared to the other C1=C3 bond. Likewise, the C3–C4 single bonds in both II and IV have acquired slightly double bond characters. Consequently, their experimental values [22,30] were shortened by 0.079 and 0.024 Å, compared to those of ethylidenimine [46] and methyl cyanide [47] respectively; (2) The delocalization energies of II and IV are comparable and have grand totals of 61.12 and 67.41 kcal/mol respectively. These rough estimates confirmed the unequivocal preference of IV over II by 6.29 kcal/mol; (3) The vicinal N lone pair interaction with C3–C4 antibond (*n*_N_ → σ*_C3C4_) favours II by 0.77 kcal/mol; (4) Additionally, II benefits from the geminal σ_C3C4_ → σ*_C1C3_ interaction (9.64 kcal/mol); while IV profits from the vicinal π_C6N7_ → π*_C1C4_ interaction (9.89 kcal/mol). The outcome of these interactions favours IV by 0.25 kcal/mol; (5) Therefore, the provenance of preference of IV over II, stems from the two geminal σ_C3C4_ → σ*_C4N6_ and σ_C4N6_ → σ*_C3C4_ interactions that collectively stabilized IV by 9.43 kcal/mol and II by only 2.71 kcal/mol; (6) The comparatively powerful delocalization interactions of III, with a grand total of 99.22 kcal/mol, might have led to its high reactivity and hence less stability compared to II and IV; (7) The strongest delocalization energy of TS1 that involves the interaction between the C3–H2 bond (σ_C3H2_) with C3N4 anti bond (σ*_C3N4_) has stabilized it by 119.30 kcal/mol. Likewise, σ_C1-H7_ → n*_C3_ is the strongest hyperconjugative interaction in TS2 with a value of 269.74 kcal/mol. These extremely powerful delocalization interactions have led to the weakness of the C3–H2 and C1–H7 bonds and hence drove them to dissociate forming the migrating protons.

**Table 7:** Second order perturbation (*E*(2)) estimation of the hyperconjugative energies (kcal/mol) ^a^ of Z-prop-2-ynylideneamine (II), intermediate (III), vinyl cyanide (IV), and Transition States (TS1 and TS2) which were calculated using B3LYP/aug-cc-pvdz level of theory.

Interaction	II	III	IV	Interaction	TS1	TS2
σ_C1-H2_ → σ*_C1-C3_(σ*_C4-H5_)	5.62	6.50	(5.13)	σ_C1-C5_ → σ*_C3-N4_	5.93	6.54
σ_C1-H2_ → σ*_C3-C4_	6.62	3.72	6.38	π_C1-C5_ → π*_C3-N4_	25.16	53.53
σ_C1-C3_ → σ*_C3-C4_	6.31	11.74	<0.50	σ_C3-H2_ → σ*_C3-N4_	119.30	<0.50
σ_C1-C3_ → σ*_C4-N6_	0.67	5.68	5.28	_σC1-H7_ → n*_C3_	<0.50	269.74
π_C1-C3_ → π*_C4-N6_	16.02	33.45	18.11	σ_C5-H7_ → σ*_C1-C3_	7.24	11.11
σ_C3-c4_ → σ*_C1-C3_	9.64	9.04	<0.50	n_N_ → σ*_C1-C3_	11.76	10.71
σ_C3-C4_ → σ*_C4-N6_	1.22	7.33	4.84	π*_C3-N4_ → π*_C1-C5_	40.66	28.89
σ_C4-N6_ → σ*_C3-C4_	1.49	15.31	4.52	σ*_C1-N4_ → σ*_C1-C3_	30.39	<0.50
π_C6-N7_ → π*_C1-C4_	<0.50	<0.50	9.89	σ_C1-C5_ → σ*_C1-C3_	<0.50	11.28
n_N_ → σ*_C3-C4_	13.03	5.95	12.26	σ*_C1-N4_ → _σC1-N4_	10.04	<0.50
Total	61.12	99.22	67.41	Total	251.48	393.30

^a^ Threshold for printing: 0.5 kcal/mol but considered 0.50 kcal/mol when working the total.

All our theoretical investigations of the relative stability of II and IV have supported the latter by 33.3–35.5 kcal/mol in excellent agreement with a theoretical value of 33.41 kcal/mol [33]. Generally, the relative stabilities of tautomers can be approached through the steric, electrostatic or hyperconjugative interactions [48]. The relative effects of these three factors on the relative stabilities of II, III and IV were analyzed by performing NBO calculations using the $DEL Keylist of the NBO version 3.1 [49] incorporated into the Gaussian09 Suite [50]. In Table 8 are listed the total SCF, deletion and hyperconjugative energies of II, III and IV which were estimated by using RHF and B3LYP methods with 6-311++G** and aug-cc-pvdz basis sets. The combined effect of the three factors is shown by the total SCF energy; where II is favoured over III by 9.20 kcal/mol (RHF/6-311++G**), 8.84 kcal/mol (RHF/aug-cc-pvdz), 0.32 kcal/mol (B3LYP/6-311++G**) and 0.21 kcal/mol (B3LYP/aug-cc-pvdz); whilst IV is supported over II by 33.35–37.42 kcal/mol using these same modeled levels of theory. 

**Table 8:** NBO analyses of the total SCF, deletion and delocalization energies (a.u.) of Z-prop-2-ynylidemeamine (II), prop-1,2-diene-1-imine (III) and vinyl cyanide (IV) which were calculated by using HF and B3LYP methods with 6-311++G** and aug-cc-pvdz basis sets.

Level of Theory	Energy/a.u.	II	III	Δ*E*_1_^a^	IV	Δ*E*_2_^b^
RHF/6-311++G**	Total SCF energy (full)	−169.752021	−169.737351	+9.20	−169.8113786	+37.25
	Energy of Deletion (L)	−169.441285	−169.302242	+87.25	−169.5367135	+59.88
	Delocalization energy	−0.310736	−0.435109	−78.05	−0.274665	−22.63
RHF/aug-cc-pvdz	Total SCF energy (full)	−169.729776	−169.715694	+8.84	−169.7894057	+37.42
	Energy of Deletion (L)	−169.439244	−169.301429	+86.48	−169.5286064	+56.08
	Delocalization energy	−0.290532	−0.414266	−77.64	−0.260799	−18.66
B3LYP/6-311++G**	Total SCF energy (full)	−170.829746	−170.829238	+0.32	−170.8829004	+33.35
	Energy of Deletion (L)	−170.551258	−170.431655	+75.05	−170.632217	+50.80
	Delocalization energy	−0.278488	−0.397583	−74.73	−0.250683	−17.45
B3LYP/aug-cc-pvdz	Total SCF energy (full)	−170.800179	−170.799846	+0.21	−170.8534328	+33.41
	Energy of Deletion (L)	−170.500599	−170.420204	+50.45	−170.6127173	+70.35
	Delocalization energy	−0.299580	−0.379641	−50.24	−0.240715	−36.94

^a^ ∆*E*_1_ = *E*_III_ − *E*_II_ (kcal/mol); ^b^ ∆*E*_2_ = *E*_II_ − *E*_IV_ (kcal/mol).

Localized Lewis structures for II, III and IV were obtained by the energies of deletion. In these structures the steric and electrostatic interactions prevailed as the hyperconjugative ones were excluded. All our calculations of these Lewis structures showed also preferences of II over III by 50.45–87.25 kcal/mol and of IV over II by 50.80–70.35 kcal/mol. In contrast, the hyperconjugative interactions were competitive enough to support III over II by energies of 50.24–78.05 kcal/mol; and to favour II over IV by 17.45–36.94 kcal/mol. 

A global glance at all these energy values might come up with the following observations: (1) The steric effects between the hydrogen atoms and other bulky groups are minimal; (2) There is powerful electrostatic repulsion in the vicinity of the linear C=C=C=N moiety of III leading to its relatively high instability; (3) The electrostatic repulsion in II subdued the steric hindrance in IV. This has been manifested by the preference of the Lewis structure of the latter; (4) The delocalization contribution strongly supported III over II by almost a similar amount of energy leading to nearly equal total energies (0.21–0.32 kcal/mol) for the two tautomers; (5) Finally, there is no doubt that IV is more stable than II. However, the relative stabilities of II and III are still open for more rigorous investigations.

## 3. Computational Details

All calculations were performed using the Gaussian09 suite of programs [50]. The geometries of E-prop-2-ynylideneamine (I), Z-prop-2-ynylidemeamine (II), prop-1,2-diene-1-imine (III), the proposed intermediate for this tautomerization reaction, and vinyl cyanide (VI) were fully optimized to a minimum using the B3LYP functional of the Density Functional Theory (DFT), second order Møller–Plesset perturbation theory (MP2) and the Coupled Cluster with singles and doubles (CCSD) methods with 6-311++G** and aug-cc-pvdz basis sets. The Transition Sates (TS1 and TS2) of II to produce IV were requested [51], using the selected methods and basis sets. Intrinsic Reaction Coordinates (IRCs) [52] calculations were implemented for transition structures connecting minima in their potential energy surfaces. The resulting imaginary frequencies and IRCs of the displacements of the bond lengths and angles involving the atoms of interest, were visualized by GaussView [53] and Chemcraft [54] programs. 

The hyperconjugative energies and charge distribution of II, III, TS1, TS2 and IV were estimated by applying version 3.1 of the natural bond orbital (NBO) suite [49] and using B3LYP, MP2 and CCSD methods with 6-311++G** and aug-cc-pvdz basis sets. The configurational energy near-equivalence between II and III and between II and IV were lifted by analyzing their NBO energy of deletion using HF and B3LYP methods with 6-311++G** and aug-cc-pvdz basis sets. The prime purpose of this step was to investigate the relative stabilities of these substrates based on the NBO donor-acceptor approach.

## 4. Conclusions

The MP2, DFT/B3LYP and CCSD methods with 6-311++G** and aug-cc-pvdz were applied to monitor the geometries, the kinetics for the isomerization, tautomerization and relative stabilities of E-prop-2-ynylideneamine (I), Z-prop-2-ynylideneamine (II) and vinyl cyanide (IV) through prop-1,2-diene-1-imine (III) as an intermediate. All substrates were shown to have Cs symmetry and comparable geometrical parameters. They are in excellent agreement with experimental ones. All modeled levels of theory predicted higher dipole moments than experimental ones. CCSD/6-311++G** level of theory predicted the most accurate experimental dipole moment of IV of 3.92 Debye within an error of 0.51%. 

As for E-HC≡CCH=NH(I) ↔ Z-HC≡CCH=NH(II) isomerization, all applied levels of theory have shown that II is favoured by 1.707 to 3.707 kJ/mol in good agreement with the experimental value of 3.264 ± 0.837 kJ/mol. The *K* values indicated that the equilibrium concentration of II ranges from double to five times that of I. 

Our theoretical studies have indicated clearly that vinyl cyanide (IV) is favoured over Z-prop-2-ynylideneamine (II) at low temperature by total energy differences of 137.3 to 148.4 kJ/mol. At high temperature, a reverse reaction becomes spontaneous and II is preferred. This could be taken as indication that Z-prop-2-ynylideneamine (II) might be present in hot core forming stars. The values of *K* gauge the huge equilibrium concentration of IV compared to that of II. This fact was taken as a confirmation of the high ISM abundance of vinyl cyanide and the equivocal existence of Z-prop-2-ynylideneamine in space. The provenance of IV overall preference was found to be mainly due to the two geminal σ_C3C4_ → σ*_C4N6_ and σ_C4N6_ → σ*_C3C4_ interactions. 

The MP2 and CCSD methods predicted Z-prop-2-ynylideneamine (II) to be more stable than prop-1,2-diene-1-imine (III) by not less 6 kcal/mol; whilst B3LYP have straitened this gap to 0.21 kcal/mol in favour of II. The competitiveness of III was attributed to the strong hyperconjugative interactions. A close look at the relative stabilities of II and III, using other DFT functionals, might be necessary for any future investigation of this matter. 
